# Explant technique for the isolation of stem cell from the dental pulp of permanent teeth

**DOI:** 10.1186/1753-6561-8-S4-P66

**Published:** 2014-10-01

**Authors:** Eduarda GZ Centeno, Camila P Ferrúa, Fernanda Nedel, Sandra BC Tarquinio, Flávio F Demarco

**Affiliations:** 1 Nucleus of Cellular and Tecidual Biology (NCTBio), Federal University of Pelotas, Pelotas, RS, Brazil

## Background

Currently, the use of stem cells from permanent (DPSCs) and deciduous teeth (SHEDs) has enabled many therapeutic advances. Despite numerous sources of stem cells, DPSCs and SHEDs have shown several advantages, since the isolation method is noninvasive and cells present rapid expansion *in vitro *[1]. DPSCs have shown the capacity to form a dentin-pulp like complex. Due to its high osteogenic potential, these cells have been applied around dental implants in order to form new bone tissue [2-4]. Given the clinical relevance in developing research towards DPSCs, this study aimed to isolate stem cells from dental pulp tissue using the explant technique.

## Methods

This study was approved by the Ethics Committee in Research of the Faculty of Dentistry, Federal University of Pelotas (FO-UFPel). Third molars were collected immediately after extraction, stored in culture medium and kept at low temperature to be transported to the laboratory. Fracture lines were made in the teeth with a chisel, in order to expose the pulp. The tissue was removed from the pulp chamber and fragmented into explants which were placed in 6 well plates and DMEM/Ham F12 medium with 15% of FBS (Hyclone), 1% of antibiotic and 1% of aminoacids non essential (Gibco) was added. The plates were incubated, for 14 days and analyzed using an inverted microscope.

The viability of the DPSCs was determined by measuring the reduction of soluble MTT [3-(4,5-dimethylthiazol-2-yl)-2,5- diphenyltetrazolium bromide] to water-insoluble formazan [4]. Cells in the 3°, 4°, 5° and 6° passages were used [5]. Briefly, cells were seed at a density of 2500 cells/cm2 and 1000 cells/cm2 and incubated at 37°C in a humidified atmosphere of 5% CO2/95% air for 1, 3, 5 and 7 days. After incubation the medium was removed and 180 μL of DMEM/Ham F12 and 20 μL MTT (5 mg MTT/mL solution) was added to each well. The plates were incubated for an additional 4 h and the medium was discarded. 200 μL of DMSO was added to each well, and the formazan was solubilized on a shaker for 5 min at 100 rpm. The absorbance of each well was read on a microplate reader (Thermo TP-plate reader, Thermo Fisher Scientific, Waltham, MA, USA) at a wavelength of 450 nm. All observations were performed in triplicate and validated by at least two independent experiments.

## Results and conclusion

Cell migration, morphology and proliferative results were adequate and compatible with DPSC isolation. Cell presented a fusiform morphology and an elongated cytoplasm, indicating high cell metabolism. The cellular viability assay showed that cultures seeded with 2500 cells/cm² exhibit higher values of absorbance compared to 1000 cells/cm² cell density. In addition, the increase in cell incubation time improved absorbance values. In summary, after 14 days of explant culture, there was an adequate cell migration and proliferation, indicating the isolation of DPSCs using the explant technique.
